# Lutein isolated from *Scenedesmus obliquus* microalga boosts immunity against cyclophosphamide-induced brain injury in rats

**DOI:** 10.1038/s41598-022-25252-9

**Published:** 2022-12-30

**Authors:** Farouk K. El-Baz, Abeer Salama, Sami I. Ali, Rania Elgohary

**Affiliations:** 1 Plant Biochemistry Department, National Research Centre (NRC), 33 El Buhouth St. (Former El-Tahrir St.), Dokki, Cairo, 12622 Egypt; 2 Pharmacology Department, National Research Centre (NRC), 33 El Buhouth St. (Former El-Tahrir St.), Dokki, Cairo, 12622 Egypt; 3 Narcotics, Ergogenics and Poisons Department, National Research Centre (NRC), 33 El Buhouth St. (Former El-Tahrir St.), Dokki, Cairo, 12622 Egypt

**Keywords:** Biochemistry, Immunology, Plant sciences

## Abstract

Lutein is a naturally potent antioxidant carotenoid synthesized in green microalgae with a potent ability to prevent different human chronic conditions. To date, there are no reports of the immune-stimulating effect of pure lutein isolated from *Scenedesmus obliquus.* Thus, we isolated the natural lutein from *S. obliquus* and evaluated its effectiveness as an immunostimulant against cyclophosphamide-induced brain injury. We purified all-E-(3R, 3′R, 6′R)-Lutein from *S. obliquus* using prep-HPLC and characterized it by ^1^H- and ^13^C-NMR spectroscopy. We assigned rats randomly to four experimental groups: the Control group got a vehicle for lutein dimethyl sulfoxide for ten successive days. The Cyclophosphamide group received a single i.p injection of Cyclophosphamide (200 mg/kg). Lutein groups received 50 and 100 (mg/kg) of lutein one time per day for ten successive days after the cyclophosphamide dose. Lutein administration reduced brain contents of Macrophage inflammatory protein2 (MIP2), cytokine-induced- neutrophil chemoattractant (CINC), and Matrix metalloproteinase 1 (MMP1). Besides, it lowered the contents of interleukin 1 beta (IL-1β) and interleukin 18 (IL-18), associated with low content of NLR pyrin domain protein 3 (NLRP3) and consequently caspase-1 compared to the cyclophosphamide group. In the histomorphometric analysis, lutein groups (50 and 100 mg/Kg) showed mild histopathological alterations as they significantly reduced nuclear pyknosis numbers by 65% and 69% respectively, compared to the cyclophosphamide group. This is the first study that showed the immunomodulatory roles of lutein against cyclophosphamide-induced brain injury via decreasing neuroinflammation, chemokines recruitment, and neuron degeneration with the modulation of immune markers. Hence, lutein can be an effective immunomodulator against inflammation-related immune disorders.

## Introduction

Cyclophosphamide, a cancer chemotherapy agent, stimulates malignant growth and life expectancy in cancer management. Its metabolites phosphoramide mustard and acrolein produce unfortunate symptoms^1^ besides other side effects such as hepatotoxicity, nephrotoxicity, neuronal toxicity, and immunotoxicity^2^. Growing evidence indicates the neurotoxicity of cyclophosphamide^3^ through inflammatory cytokines^4^. Metabolite acrolein induces lipid peroxidation and reactive oxygen species (ROS) release causing cellular impairments^5^. Interleukin 1 beta (IL-1β) and interleukin 18 (IL-18) cytokines provoked innate immunity and inflammation^6^. The production of IL-1β and IL-18 by the innate immune system is involved in experimental brain injury^7^. They induce CINC-1 and MIP-2 chemokines production and boost neutrophil propagation in the brain during acute and chronic injury^8^. In brain injury, the destruction of the blood–brain barrier results in inflammatory signals that enter the periphery through the blood and provoke a systemic immune response. These inflammatory signals initiates the repair tissue damage or necrosis and stimulate nerve and blood vessel regeneration^9^.

Antioxidants showed their benefits in preventing and treating cyclophosphamide neurotoxicity. Lutein is an orange-red carotenoid pigment classified as the main xanthophyll component produced by plants especially marigold flowers that are used for the sustainable production of lutein in the market^10^. However, the low content (0.03%) of the lutein, and cultivation time, besides the labor-intensive separation process of marigold petals are big obstacles to this commercial production^11^. Microalgae are a promising renewable alternative source of lutein production and other carotenoids^10^. Microalgae such as Scenedesmus sp.^12^, *Chlorella zofingensis*^13^, *Muriellopsis sp.*^14^, *Parachlorella kessleri*^15^, and *Chlorella protothecoides*^16^ with their high growth rate, renewable biomass production, and high content of lutein can provide the needs of lutein commercial production compared to marigold and other leafy vegetables^17^. S. *obliquus* is one of the promising microalgae producing a valuable content (4–4.52 mg/g) of lutein^18^. Lutein is a forty carbons xanthophyll containing a specific series of leading, conjugated double bonds besides two hydroxyl groups one on each side of the molecule. This chemical structure improves its capacity to scavenge free radicals and singlet oxygen and increases its biological effectiveness^19,20^. The effective antioxidant, anti-inflammatory, and other therapeutic properties of lutein have increased its use in pharmaceutical, cosmetic, and nutraceutical applications^21^. It is an energetic constituent that can accumulate in the human retina in the macula lutea, so it is an important factor in protecting against the visual loss associated with age-related macular degeneration^22^. Moreover, can inhibit different chronic diseases including cardiovascular diseases, cancers, age-related diseases, diabetes, retinopathy, and atherosclerosis. It can also protect the skin cells against UV injury^23^. Lutein is a natural antioxidant has a potent ability to inactivate the singlet oxygen, capture hydroxyl radicals, bind to lipids to suppress lipid oxidation, and inhibit the free radical injury to bio-membranes^24^. It exerts its immunomodulatory effect through its antioxidative properties and controls the immune response via modifying cytokines and other immune mediators expressions^25^. Therefore, the current study aims to isolate and identify the lutein from *S. obliquus* using prep-HPLC and ^1^H- and ^13^C-NMR. Moreover, the immunomodulatory effect of lutein against cyclophosphamide-induced brain injury via the modulation of brain contents of MIP2, CINC, MMP1, IL-18, IL-1β, NLRP3, and caspase-1 was investigated in this study.

## Materials and methods

### Chemicals

For extraction and column chromatography separation, n-hexane, acetone, methanol (analytical grade), and silica gel (40–63 μm) from Sigma-Aldrich (USA) were used. Acetone and n-hexane (HPLC grade) from Sigma-Aldrich (USA) were used for HPLC analysis and purification. Furthermore, deuterated chloroform from Merck (Darmstadt, Germany) was used for NMR measurements.

### Cultivation of *S. obliquus*

*S. obliquus* was isolated from the freshwater community of the River Nile in October 2011 and grown on BG11 media^26^**.** Cultivation was conducted in 17 L capacity plastic bottles having 15 L of *S. obliquus* culture with the following conditions: continuous aeration, culture temperature of 22 ± 3 °C, and constant light intensity ≈of 2500 lx using fluorescent light. After 10 days of algal growth, the culture was transferred to a fully automated and computer-controlled vertical photobioreactor with a capacity of 4000 L. Carbon dioxide was injected into the culture as a carbon source. The culture was left to grow until the biomass reached 2–2.5 g/L. The biomass of *S. obliquus* was harvested by centrifugation at 2000 rpm for 15 min using a basket centrifuge. Samples were washed twice with water, dried in an oven at 50 °C, ground into a homogenous powder, and stored in a deep freezer until used.

### Preparation of lutein extract

The fine powder of *S. obliquus* (300 g) was soaked in 1.5 L of hexane: acetone (1:1, v/v) in a 5 L conical flask and kept on an orbital shaker (Stuart, England) at 160 rpm at room temperature for 24 h. Then, the extract was centrifuged (Sigma 3–18ks Centrifuge, Germany) at 5000 rpm for 20 min at 25 °C to separate cell debris from the supernatant. The extraction step was repeated twice using the freshly prepared solvent mixture, and the pooled supernatants were concentrated using a vacuum rotary evaporator (Heidolph Unimax 2010, Germany) at 40 °C to dryness giving the *S. obliquus* hexane: acetone crude extract (SOCE). All the extraction steps were performed in dim light^27^**.**

### HPLC analysis

The HPLC analysis of SOCE and its fractions were performed using an Agilent 1260 infinity series HPLC–DAD system (Agilent Technologies, Waldbronn, Germany) equipped with a binary gradient Agilent 1260 prep pump (G1361A) and an autosampler Agilent 1260 prep ALS (G2260A). Agilent diode array detector 1260 DAD VL (G1315D) was employed for the detection of carotenoids. The separation was performed using an Agilent normal phase (NP) silica column (ZORBAX RX-Sil, 5 µm, 4.6 × 150 mm). The following solvents (A) n-hexane and (B) acetone were used at a flow rate of 1 mL/min using a gradient between solvents A and B following the method of Prum et al.^28^ with some modifications as follows: B was run at 0 to 30% for 5 min, 30 to 50% for 15 min, 50 to 100% for 3 min, and maintaining 100% of B until the end of the separation at 30 min. The peaks were integrated at 450 nm.

### Purification of lutein

The HPLC chemical profile of SOCE showed an abundant peak (P1) at 8.89 min representing about 40% of the total peak area. Consequently, to isolate the compound corresponding to this peak, a portion of SOCE (8 g) was fractionated by Vacuum Liquid Chromatography (VLC) over Sigma-Aldrich silica gel (40–63 mesh, 250 g) and eluted with n-hexane (1L), n-hexane/EtOAc (95:5, 1.5L), n-hexane/EtOAc (70:30, 2L), EtOAc (1L), and EtOAc/MeOH (50:50, 1L) to yield 4 fractions namely, F1 (1.2276 g), F2 (2.8654 g), F3 (0.3598 g), and F4 (2.7962 g). The two solvent systems of n-hexane and n-hexane/EtOAc (95:5) were collected in one batch, while in other solvent systems, each system was collected in one batch. The HPLC analysis revealed that F3 is the rich fraction of the compound corresponding to P1. F3 (orange-red precipitate) was further purified by Medium Pressure Liquid Chromatography (MPLC) using a BUCHI GlasPure glass chromatography column (ID: 230 × 36 mm), which was packed with 125 g dry silica gel (40–63 mesh) and wetted by 1000 mL of hexane using Lab alliance series 1 pump (USA). Then F3 (0.3598 g) was dissolved in ethyl acetate and loaded on the column using an injection column (BUCHI, Switzerland) and eluted at a flow rate of 4 ml/min by n-hexane (150 mL), n-hexane/EtOAc (95:5, 400 mL), n-hexane/EtOAc (70:30, 500 mL), n-hexane/EtOAc (30:70, 300 mL), and EtOAc (700 mL) to yield 4 subfractions, FI (0.0721 g), FII (0.1061 g), FIII (0.1641 g), and FIV (0.0161 g) based on HPLC profile. The HPLC analysis revealed that FIII is a semi-pure compound. Then FIII (0.1641 g) was entirely purified by using preparative HPLC (Agilent 1260 infinity series) using an Agilent prep silica column (Agilent 5 Prep-Silica, 150 × 21.2 mm). The isocratic mobile phase (20% acetone in hexane) at a flow rate of 20 ml/min was performed to give pure compound (lutein, 127 mg) at 11.809 min as an amorphous orange-red powder.

### Identification of lutein from *S. obliquus* by nuclear magnetic resonance (NMR)

The ^1^H- and ^13^C-NMR (500 MHz, 125 MHz) spectra of P1 (isolated from *S. obliquus*) were recorded on an NMR spectrometer (JEOL, USA) with CDCl_3_ as the solvent. The chemical shifts are reported in ppm (parts per million; δ) and coupling constants (J) are expressed in Hz. TMS was used as an internal standard. For improving the signal-to-noise ratio, total scans of 128 and 725 were performed for ^1^H- and ^13^C-NMR, respectively. The data were analyzed using the software program MestReNova v8.0.2 (2012 Mestrelab Research S. L.).

### Animals

Adult male Wister albino rats aged 4–6 weeks (120–140 g) were purchased from the animal house colony of the National Research Centre (Dokki, Cairo, Egypt). Animals were kept in standard cages, under pathogen-free conditions, and maintained under controlled room temperature and normal dark–light cycles. Animals were provided with standard food and water ad libitum. Rats were allowed to adapt to these conditions for 2 weeks before beginning the experimental protocol. All experimental procedures were conducted according to the ethical principles and guidelines of the use, care, and handling of experimental animals adopted by the Medical Research Ethics Committee at the National Research Centre, Egypt, and approved under (Reg. No. 19/116), which is based on the Principles of Laboratory Animal Care (NIH No. 85:23 revised 1985). All experimental procedures were conducted in compliance with the Animal Research: Reporting of In Vivo Experiments (ARRIVE) guidelines.

### Kits and chemicals

Cyclophosphamide was obtained from Santa Cruz Biotechnology, Inc. (California, USA). Interleukin-18 (IL-18; SL0400Ra), Interleukin-1β (IL-1β; SL0402Ra), Macrophage inflammatory protein2 (MIP2; SL0465Ra), cytokine-induced- neutrophil chemoattractant (CINC; SL1588Ra), Matrix metalloproteinase 1 (MMP1; SL0480Ra), NLR pyrin domain protein 3 (NLRP3; SL1497Ra) and caspase-1 (SL1601Ra) were determined using ELISA kits (Sunlong Biotech Co., Ltd, China). All other chemicals used in this study are of the highest grade commercially available.

### Experimental design

Adult male Wister albino rats were randomly assigned to four experimental groups each having 8 animals and treated as follows:

1. Control group where rat received vehicle for lutein (DMSO) for 10 consecutive days.
2. Cyclophosphamide group where rats were intraperitoneally injected with a single injection (200 mg/kg)^29^.
3. Lutein group where rats were administered lutein (50 mg/kg)^30^ once daily for 10 consecutive days after cyclophosphamide injection.
4. Lutein group where rats were administered lutein (100 mg/kg)^30^ once daily for 10 consecutive days after cyclophosphamide injection.

### Tissue samples

At the end of the experiment, the animals were sacrificed by decapitation, and the brain of each rat was immediately dissected out, washed with ice‐cooled physiological saline, and homogenized in phosphate-buffered saline (pH 7.4) at 20% (w/v) for the biochemical measurements^31^. The other brain was kept in 10% formalin for histopathological assessment.

### Biochemical analysis of IL-18, IL 1β, MIP2, CINC, MMP1, NLRP3, and caspase-1

Brain contents of IL-18, IL 1β, MIP2, CINC, MMP1, NLRP3, and caspase-1 were determined using an ELISA kit (SunLong Biotec Co., LTD, China, Glory Science, and NOVA, Beijing, China). Standards and samples were pipetted into wells with immobilized antibodies specific for IL-18, IL-1β, MIP2, CINC, MMP1, NLRP3, and caspase-1 then were incubated for 30 min at 37 °C. After incubation and washing, horseradish peroxidase-conjugated streptavidin was pipetted into the wells and incubated for 30 min at 37 °C, then washed once again. Tetramethyl benzidine (TMB) substrate solution was added to the wells and incubated for 15 min at 37 °C; a color was developed proportionally to the amount of IL-18, IL-1β, MIP2, CINC, MMP1, NLRP3, and caspase-1 bound. Color development was discontinued (stop solution) and after 10 min color intensity was measured at 450 nm^32^.

### Histological examination

The dissected brains of diverse groups were fixed in 10% formalin. Fixation for one or two days was followed by dehydration in ascending grades of alcohol (70%, 90%, and three changes in absolute alcohol), clearance with xylene, impregnation in three successive changes in soft paraffin at 50 °C, and finally embedded in paraffin wax to obtain solid blocks having the tissue. Serial transverse sections of 7 μm thick were cut. Paraffin sections were mounted on glass slides covered by albumin glycerin and then stained with Haematoxylin and Eosin. Hematoxylin and Eosin sections were evaluated qualitatively under light microscopy^33^. A semiquantitative scoring system, ranging between zero and three, was used for grading both the histopathological changes. (Nuclear pyknosis) in the brain tissues of all histological samples. The scores were derived using light microscopy and scored in four categories based on the intensity of alterations: 0, absent; 1, mild; 2, moderate; 3 severe^34^.

### Statistical analysis

All the values are presented as means ± standard error of the means (SE) of (n = 8) for each group. Data of this study were evaluated by one-way analysis of variance followed by Tukey’s multiple comparisons test. Graph pad Prism software, version 5 (Inc., San Diego, USA) was used to conduct these statistical tests. The difference was considered significant when *P* < 0.05.

## Results

### Identification of lutein

**Figure 1:**
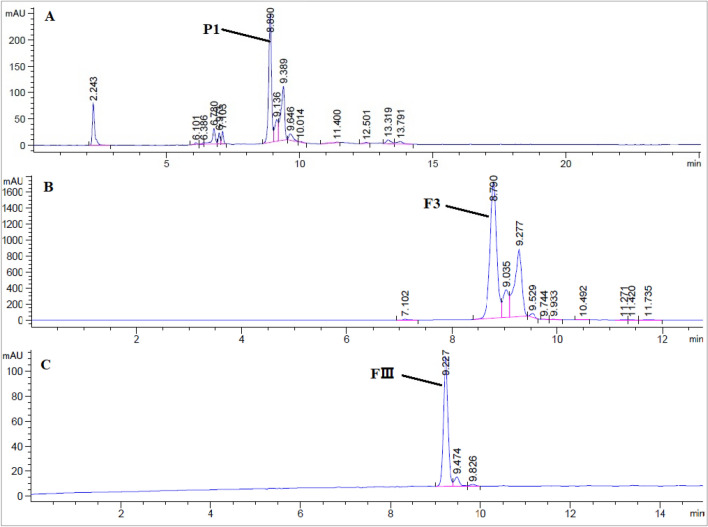
The HPLC profile of (**A**) *S. obliquus* crude extract (SOCE), (**B**) VLC fraction (F3), and (**C**) MPLC subfraction (FIII) on the silica column (ZORBAX RX-Sil, 5 µm, 4.6 X 150 mm), integration at 450 nm.

**Figure 2:**
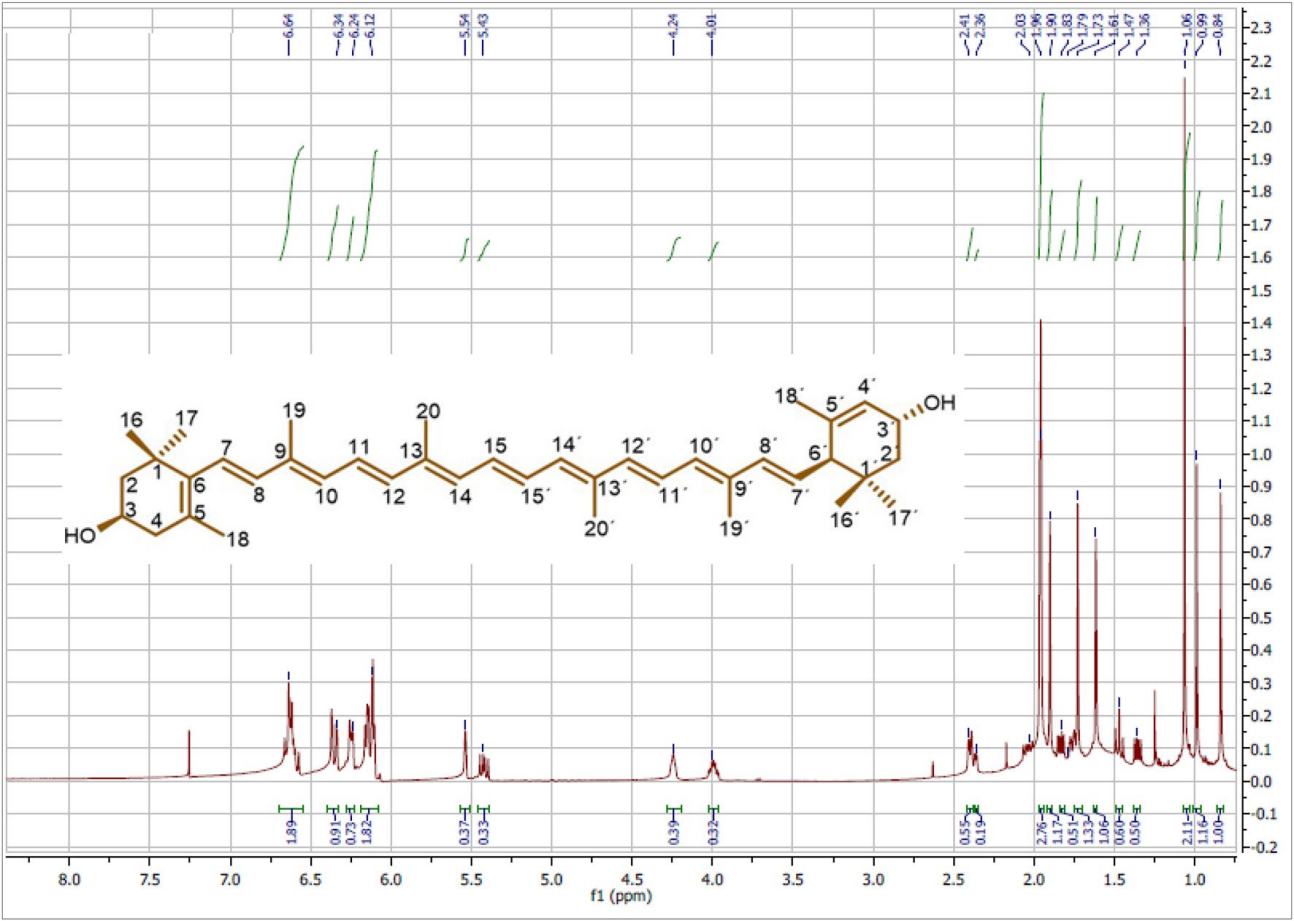
The ^1^H NMR spectrum of Lutein isolated from *S. obliquus*. (500 MHz, CDCl_3_).

**Figure 3:**
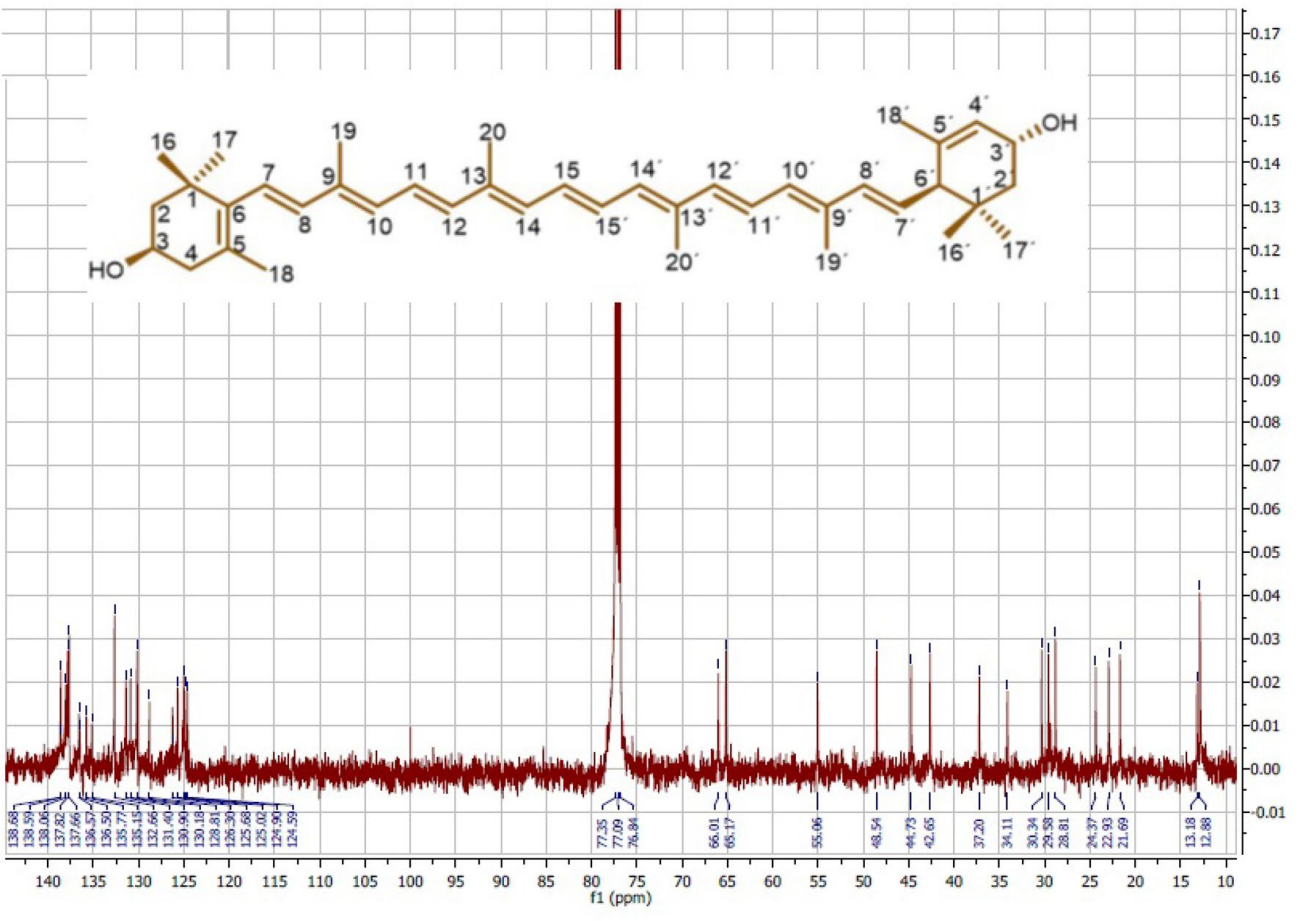
The ^13^C NMR spectrum of Lutein isolated from *S. obliquus* (125 MHz, CDCl_3_).

The extraction of carotenoids from *S. obliquus* by hexane: acetone (1:1, v/v) yielded an abundant extract yield of 22.05%. The HPLC profile of SOCE on the silica column using the mobile phase of hexane and acetone showed an abundant peak (P1) at 8.89 min with about 40% of the total area (Fig. 1A). The different chromatographic techniques (VLC and MPLC) were processed to isolate and purify the compound corresponding to P1and they revealed the VLC fraction (F3) as the rich fraction of P1 (Fig. 1B), then the MPLC sub-fraction (FIII) as a semi pure compound corresponding to P1 (Fig. 1C). The compound corresponding to P1 was entirely purified by the prep HPLC as orange-red powder and identified based on the ^1^H- and ^13^C-NMR data as Lutein (Table 1, and Figs. 2 and 3).

**Table 1:** The ^1^H and ^13^C NMR spectroscopic data of lutein isolated from *S. obliquus*.

Position	δ_H_ (500 MHz, J values in Hz)	δ_C_ (125 MHz), type
1	–	37.2, C
2	1.36 dd J = 6.61, 12.83 Hz, *1.77 ddd J = 1.51, 3.25, 6.76 Hz	48.54, CH_2_
3	4.00 m	65.17, CH
4	2.04 dd J = 9.71, 16.81 Hz, *2.36 dd J = 4.8, 5.48 Hz	42.65, CH_2_
5	–	126.3, C
6	–	138.05, C
7	6.13 m	125.68, CH
8	6.13 m	138.68, CH
9	–	135.15, C
10	6.15 m	130.9, CH
11	6.63 m	125.02, CH
12	6.35 m	137.66, CH
13	–	136.57, C
14	6.24 m	132.66, CH
15	6.63 m	130.18, CH
16	1.06 s	30.34, CH_3_
17	1.06 s	28.81, CH_3_
18	1.73 s	21.69, CH_3_
19	1.96 s	13.18, CH_3_
20	1.96 s	12.88, CH_3_
1′	–	34.11, C
2′	1.46 dd J = 4.18, 11.71 Hz, *1.83 dd J = 5.58, 12.78Hx	44.73, CH_2_
3′	4.24 s	66.01, CH
4′	5.54 s	124.59, CH
5′	–	137.82, C
6′	2.40 d J = 8.60	55.06, CH
7′	5.43 dd J = 5.07, 10.37 Hz	128.81, CH
8′	6.13 m	138.68, CH
9′	–	135.77, C
10′	6.15 m	131.4, CH
11′	6.63 m	124.9, CH
12′	6.35 m	137.82, CH
13′	–	136.5, C
14′	6.24 m	132.66, CH
15′	6.63 m	130.9, CH
16′	0.84 s	29.58, CH_3_
17′	0.99 s	24.37, CH_3_
18′	1.61 s	22.93, CH_3_
19′	1.90 s	13.18, CH_3_
20′	1.96 s	12.88, CH_3_

*These assignments may be interchanged.

### Lutein reduced brain contents of IL-18, IL-1β, and MMP1

**Figure 4:**
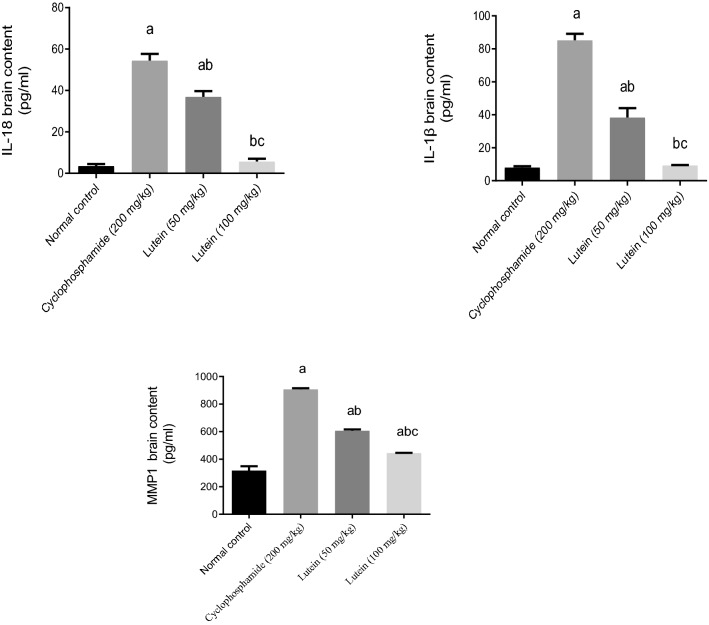
Effects of lutein on IL-18, IL 1β, and MMP1 brain contents. ^a^Significant compared to the control group. ^b^Significant compared to the Cyclophosphamide group. ^c^Significant compared to Lutein 50 group at *P* < 0.05.

Brain injury induced by cyclophosphamide produced a significant increase in IL-18, IL 1β, and MMP1 brain contents by 14-fold, tenfold, and twofold respectively, as compared to the normal control group. The 50 and 100 (mg/kg) of lutein lowered the content of IL-18 by 32% and 90%, IL-1β by 55% and 89%, and MMP1 by 33% and 51% respectively, in the brain compared to the cyclophosphamide group. Treatment with a high dose of lutein returned IL-18 and IL-1β brain contents to their normal contents (Fig. 4).

### Lutein reduced brain contents of MIP2 and CINC

**Figure 5:**
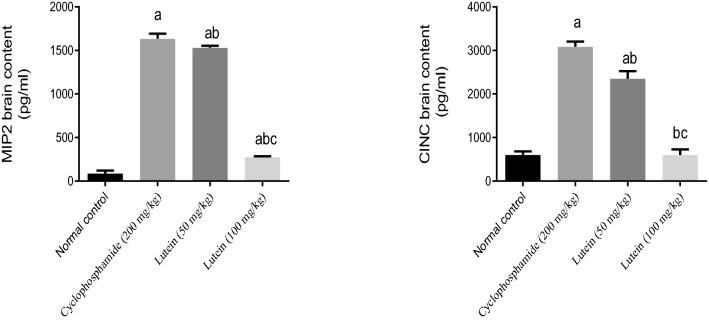
Effects of lutein on MIP2 and CINC brain contents. ^a^Significant compared to the control group. ^b^Significant compared to the Cyclophosphamide group. ^c^Significant compared to Lutein 50 group at *P* < 0.05.

Cyclophosphamide elevated MIP2 and CINC brain contents by 18-fold and fourfold compared to the control group. The 50 and 100 (mg/kg) of lutein lowered the content of MIP2 by 6% and 83% and CINC by 24% and 81% respectively, in the brain compared to the cyclophosphamide group. The high dose of lutein returned elevated the content of CINC in the brain to its normal value (Fig. 5).

### Lutein reduced brain contents of NLRP3 and caspase 1

**Figure 6:**
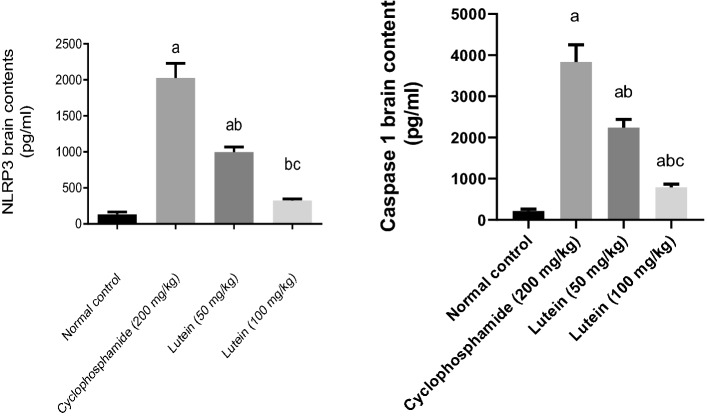
Effects of lutein on NLRP3 and caspase 1 brain contents. ^a^Significant compared to the control group. ^b^Significant compared to the Cyclophosphamide group. ^c^Significant compared to Lutein 50 group at *P* < 0.05.

Injection of Cyclophosphamide increased the content of NLRP3 and caspase 1 in the brain by 14-fold and 16-fold respectively, compared with the control group. The 50 and 100 (mg/kg) of lutein lowered the content of NLRP3 in the brain by 51% and 84% and decreased the content of caspase 1 in the brain by 42% and 79% respectively, compared with the Cyclophosphamide group. The high dose of lutein (100 mg/kg) returned NLRP3 brain content to its normal value (Fig. 6).

### Histopathological findings

#### The microphotographs of the cerebral cortex of rats

**Figure 7:**
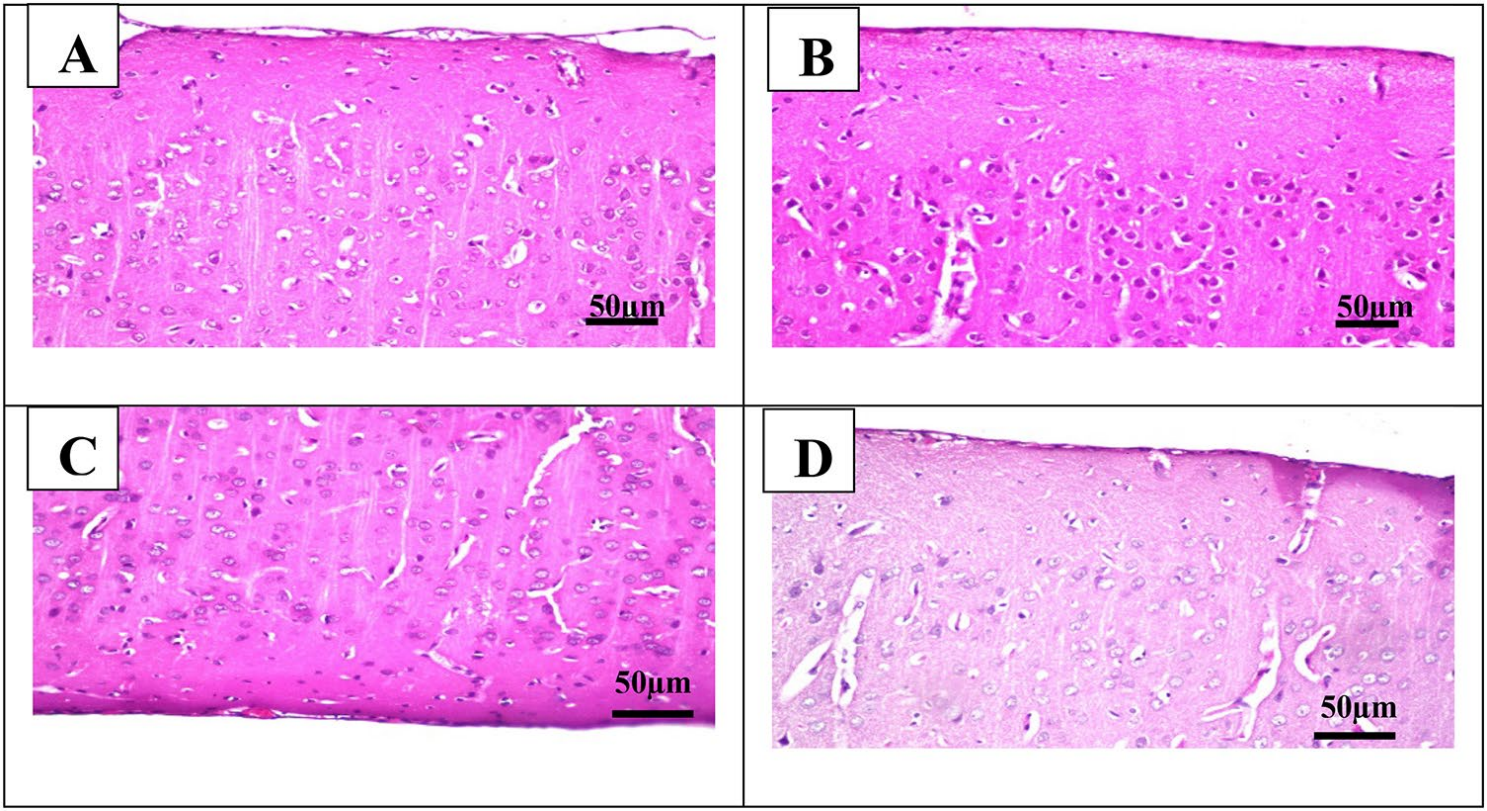
Representative photomicrographs (H&E, × 200) of the cerebral cortex **(A)** control group, **(B)** Cyclophosphamide group, **(C)** Lutein 50 group, and (**D)** Lutein 100 group.

In the control group, the examined part of the cerebral cortex showed no histopathological changes and showed ordinary histological structures of neurons (Fig. 7A). The neurons in the Cyclophosphamide group exhibited nuclear pyknosis and degeneration (Fig. 7B). Lutein 50 and 100 groups showed mild histopathological alteration (Fig. 7C,D).

### The microphotographs of the hippocampus of rats

**Figure 8:**
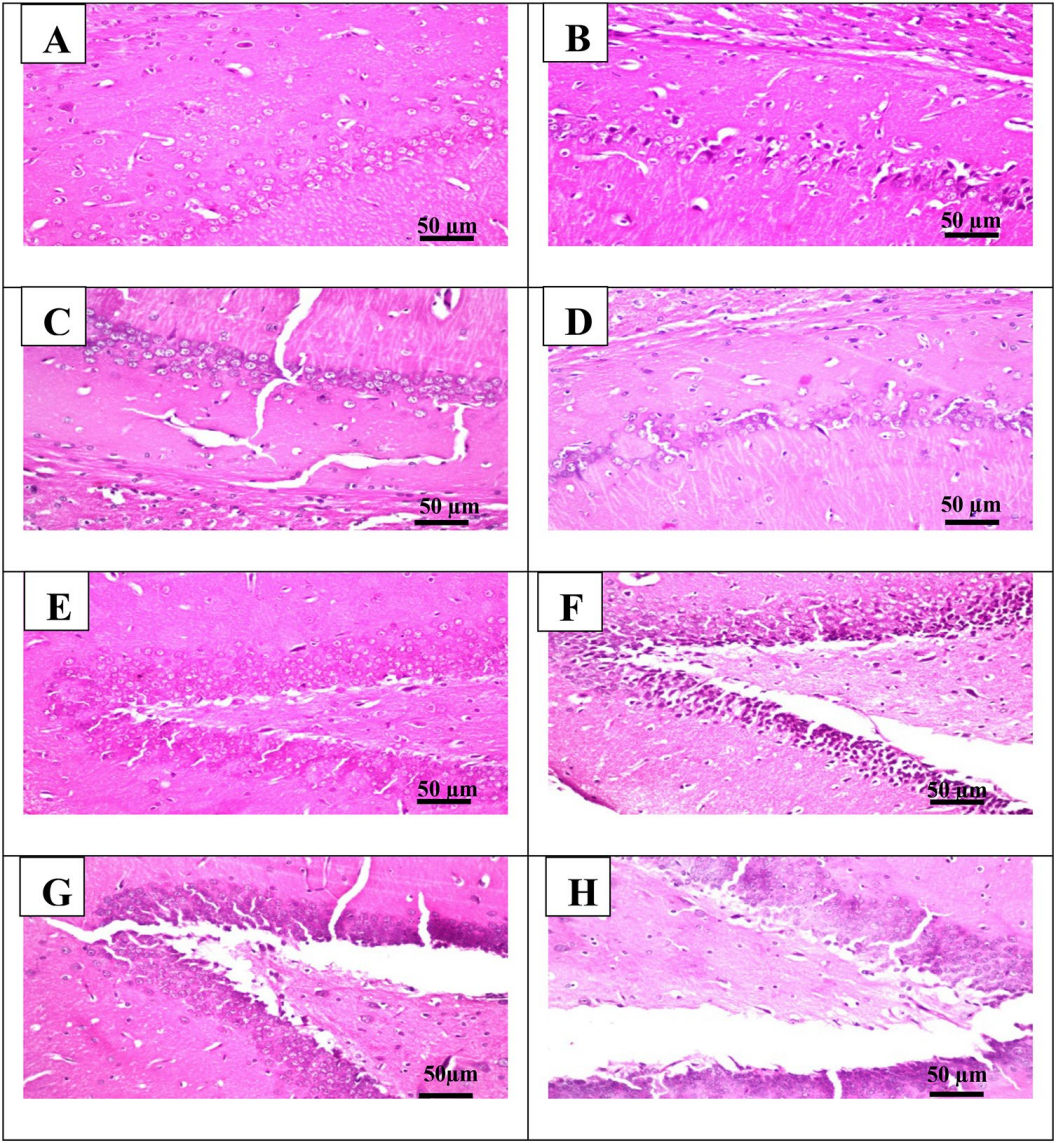
Representative photomicrographs (H&E, × 200) of **(A)** section from the subiculum of the control group, **(B)** section from the subiculum of the cyclophosphamide, **(C)** section from the subiculum of lutein 50 group, **(D)** section from the subiculum of lutein 100 group, **(E)** section from the fascia dentata and hilus of the control group, **(F)** section from the fascia dentata and hilus of cyclophosphamide group, **(G)** section from the fascia dentata and hilus of lutein 50 group, and **(H)** section from the fascia dentata and hilus of lutein 100 group.

In the control group, the examined part of the subiculum showed no histopathological changes and showed ordinary histological structures of neurons (Fig. 8A). The neurons in the Cyclophosphamide group exhibited nuclear pyknosis and degeneration (Fig. 8B). Lutein 50 and 100 groups showed no histopathological alteration (Fig. 8C,D).

In the control group, the examined part of the fascia dentata and hilus showed no histopathological changes and showed ordinary histological structures of neurons (Fig. 8E). The neurons in the Cyclophosphamide group exhibited nuclear pyknosis and degeneration (Fig. 8F). Lutein 50 and 100 groups showed no histopathological alteration (Fig. 8G,H).

### The microphotographs of the striatum of rats

**Figure 9:**
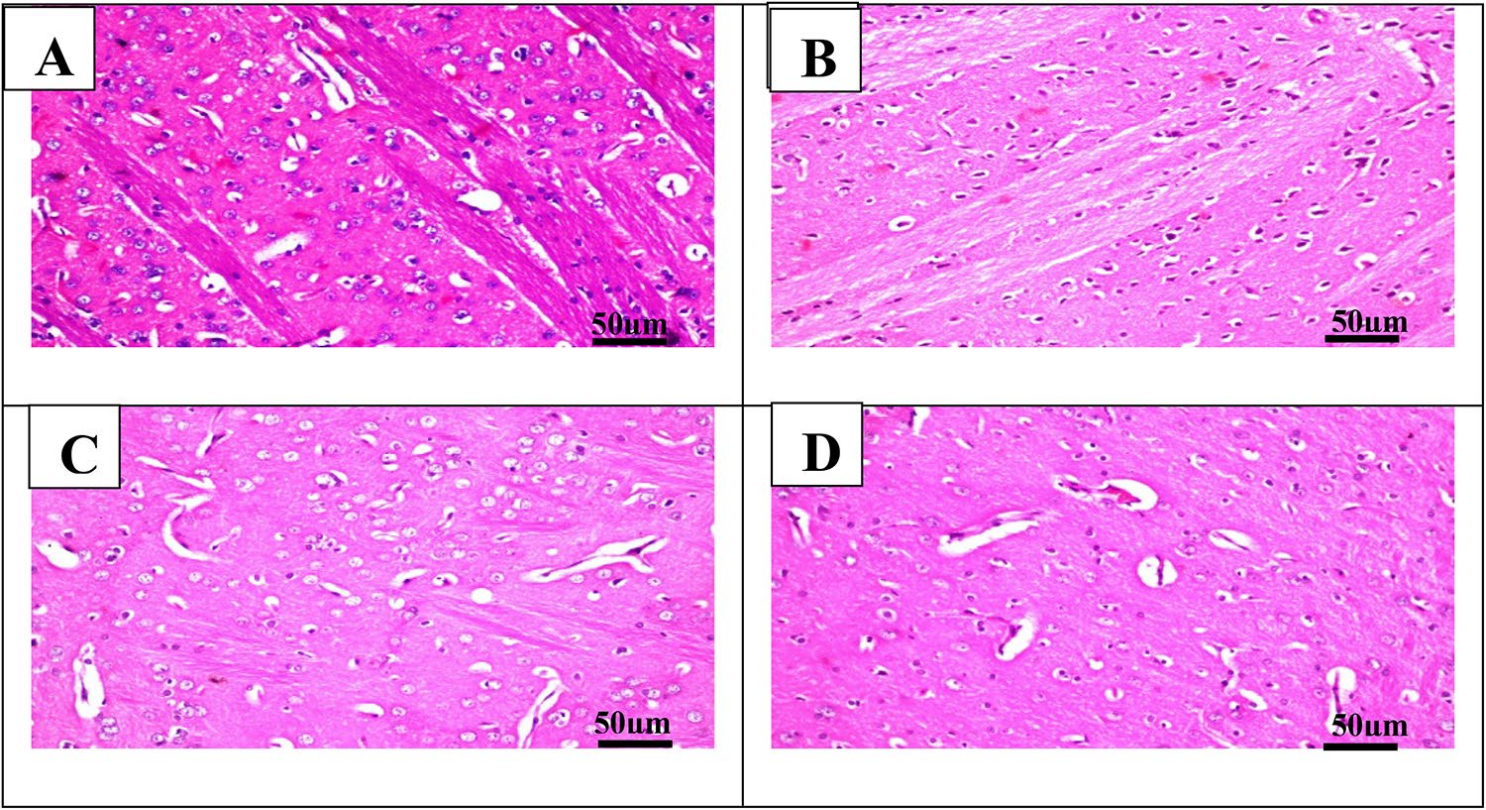
Representative photomicrographs (H&E, × 200) of the section from the striatum of **(A)** control group, **(B)** Cyclophosphamide group, **(C)** Lutein 50 group, and **(D)** Lutein 100 group.

In the control group, the examined part of the striatum showed no histopathological changes and showed ordinary histological structures of neurons (Fig. 9A). The neurons in the Cyclophosphamide group exhibited damage, nuclear pyknosis, and degeneration (Fig. 9B). Lutein 50 and 100 groups showed mild histopathological alteration (Fig. 9C,D).

### The histomorphometric analysis

**Figure 10:**
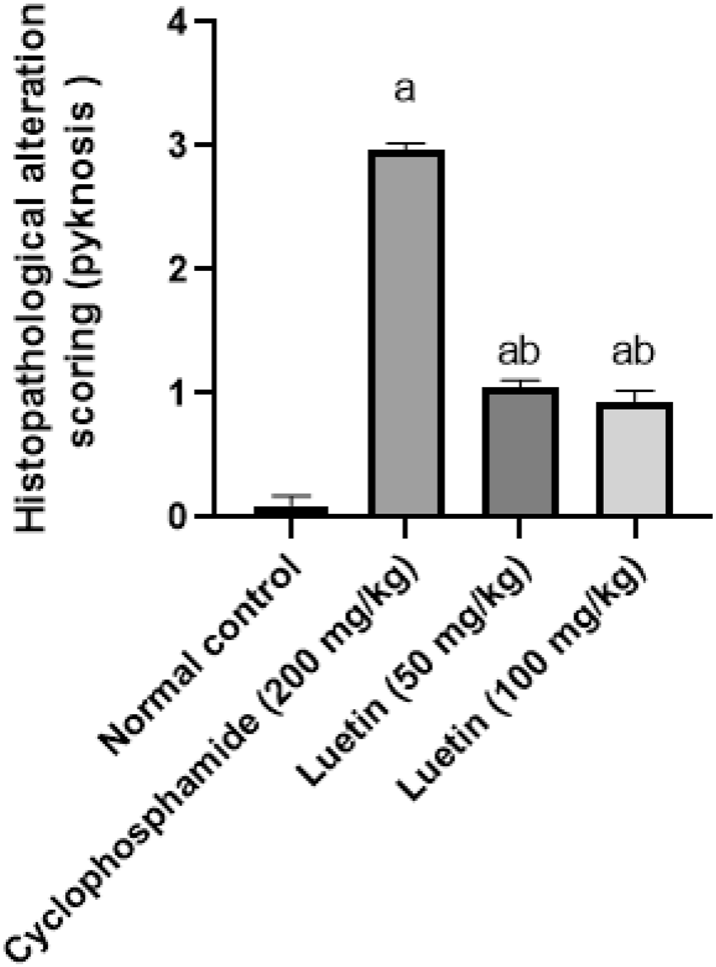
Effects of lutein on the histomorphometric analysis. ^a^Significant compared to the control group. ^b^Significant compared to the cyclophosphamide group at *P* < 0.05.

The control group revealed no histopathological alterations as it scored the lowest number of nuclear pyknosis (near to scoring 0). The cyclophosphamide group revealed severe histopathological alterations (scoring 3) as it scored the highest number of nuclear pyknosis. Interestingly, lutein 50 and 100 groups significantly showed mild histopathological alterations (scoring 1), as it reduced nuclear pyknosis numbers by 65% and 69% respectively, compared to the cyclophosphamide group (Fig. 10).

## Discussion

The lutein was purified from *S. obliquus* as orange-red powder. The ^1^H and ^13^C NMR spectroscopic data and assignments of the isolated lutein (Table 1, and Figs. 2 and 3) conformed with the previous reports^35,36^. The ^1^H NMR data of lutein isolated from *S. obliquus* revealed the chemical shift of protons corresponding to C-4 at 2.04 and 2.36 ppm, C-4′ at 5.54 ppm, C-8 at 6.13 ppm, C-10 at 5.15 ppm, C-11 at 6.63 ppm, C-12 and 12′ at 6.35 ppm, and C-18 at 1.73 ppm. These chemical shifts were equivalent to the lutein isolated from the human plasma, it configured as all-E lutein (3R,3′R,6′R) based on the difference in the relevant δH values of C-4, 4′, 8, 10, 11, 12, 12′, and 18 protons in the all-E-lutein and 9Z-lutein and 9′Z-lutein^37^. The NMR spectra of isolated lutein in the present study showed chemical shifts of protons at 4.00, 4.24, and 2.40 ppm, along with the downfield shift of carbons at 65.17, 66.01, and 65.06 ppm for the C-3, C-3′, and C-6′ respectively. These data are congruent with that of (3R,3′R,6′R) lutein isolated from marigold oleoresin^38^. Therefore, the present NMR data of lutein isolated from *S. obliquus* supports its configuration as all-E-(3R,3′R,6′R)-Lutein.

Innate immunity is the first line of defense against infectious agents and molecules released from neuron injuries^39^. The innate immune cells such as microglia and astrocytes in the brain recognize pathogens or other inflammatory triggers and activate the inflammasome. The inflammasome stimulates proinflammatory caspases which increase the release of interleukin-1 β and IL-18 which reduce the toxins released from glial and endothelial cells and so modulate neurodegenerative processes^40^. In the present study, cyclophosphamide exhibited a negative effect on innate immunity cells microglia, and astrocytes, in the brain as it accelerated the generation of proinflammatory factors of IL-1β and IL-18. The administration of lutein controlled these immune cells through the decrease of brain contents of IL1β, and IL 18. In the earlier mouse model, cyclophosphamide-induced the expression of inflammatory mediators, cytokines, chemokines, and growth factors^41^. Cheng et al.^42^ reported that lutein protects against ischemia–reperfusion injury by modulating oxidative stress, membrane lipids peroxidation, and inflammation, and has immunomodulatory properties.

Active microglia and astrocytes produce MMPs which participate as proinflammatory mediators in the brain^43,44^. MMP-1 mediates matrix degradation as it cleaves extracellular collagen I, II, and III forming embolus and ischemic stroke^45^. During brain injury, reactive oxygen species (ROS) and inflammatory cytokines activate MMPs^46^, which accelerate the generation of IL-1β and TNF-α as inflammatory cytokines^47^. These results showed for the first time the role of immunomodulatory activity of lutein via the suppression of neuroinflammatory marker MMP1 and the inhibition of IL-1β through the modulation of the immune cells in the brain injury induced by cyclophosphamide. These results suggest the cytoprotective function of lutein which may be owing to its effect as anti-inflammatory and immunomodulatory. In a previous work, MMPS modulates neurodegeneration in rats after blast-induced mild TBI^48^. Lutein significantly suppressed MMP-1 expression in melanoma cells and dermal fibroblasts^49^.

Cytokines such as interleukin (IL)-1β generate neurons degeneration and lesion exacerbation^50^. Increasing IL-1β results in chemokine induction in the brain. These chemokines as cytokine- CINC-1 and CINC-3 (also known as MIP-2) are involved in neutrophil recruitment to the spinal cord and brain of rodents following an inflammatory challenge, stress, stroke, or in response to acute and chronic injury^51^. In the present study, lutein significantly decreased CINC-3 and MIP-2 which are increased in the cyclophosphamide-treated group. These findings suggest that part of the immunomodulatory mechanism of lutein includes the prevention of the chemokines induced by neutrophil recruitment and neuron degeneration. In another study, lutein treatment significantly reduced MIP-2 concentration in aqueous humor in rats^52^.

In recent years, there is evidence that immune response dysregulation is implicated in brain injury in animal models, increasing neurological impairment, and brain pathology. The high release of cytokines is the clearest prognostic sign of clinical results in brain injury^53^. The high expression of IL-1β in a traumatic brain injury by NLRP3 inflammatory corpuscle is one of the major components of the innate immune system^54^. It responds to damage by forming an NLRP3 intracellular inflammasome complex, in which apoptosis-associated speck-like protein (ASC) binds NLRP3 to pro-caspase-1, which activates caspase-1 that converts the proinflammatory cytokines pro-IL-1β and pro-IL-18 to their active secreted forms^55^. In the present study, lutein administration at a dose of 100 mg/Kg reduced the production of IL-1β and IL-18 as it returned the contents of IL-1β and IL-18 in the brain to their normal contents, and this was associated with the low content of caspase 1 and NLRP3 in the brain. These results accord with He et al.^56^, who reported that the inhibition of the protein complex (NLRP3, ASC, and Caspase-1) reduce the production of IL-1β and IL-18. Thus, the low contents of IL-1β and IL-18 in the lutein group might be owing to the low expression of NLRP3 and consequently Caspase-1. These results are in line with the suppressive effect of β-carotene (another carotenoid) on NLRP3 inflammasome, which attenuates gouty arthritis inflammatory responses in mouse models^57^. Different natural extracts and polyphenolic compounds, have shown anti-inflammatory effects through the inhibition of NLRP3 inflammasome, and consequently the reduction of caspase-1, IL-18, and IL-1ß^58–60^.

In the present work, the cyclophosphamide group showed severe histopathological alterations with a high score of nuclear pyknosis, besides neuron damage and deterioration of neurons of the cerebral cortex, hippocampus (subiculum and fascia dentata and hilus), and striatum area of the brain. Inline Shaibah et al.^61^ reported that cyclophosphamide-treated rats showed neurons with dystrophic degenerations including enlarged blood vessels, vacuolated and deteriorated, and neurocytes. On the other hand, treatment with lutein in this study showed mild histopathological alterations with a lower number of nuclear pyknosis compared with the cyclophosphamide group. In a previous study, lutein could lessen the destructive effects of brain damage after cerebral I/R by enhancing survival and reducing neuronal damage^62^.

## Conclusion

*Scenedesmus obliquus* is a promising sustainable renewable source of natural lutein. The all-E-(3R,3′R,6′R)-Lutein was purified from *S. obliquus* by prep-HPLC and characterized by ^1^H- and ^13^C-NMR spectroscopy. Lutein administration showed an immunomodulatory role in cyclophosphamide-induced brain injury via attenuation of pro-inflammatory mediator release, including inhibition of CINC / MIP2 /NLRP3/ caspase 1, and modulation of IL-1β, IL-18, and MMP1 contents. In addition, lutein administration improved deterioration in many neurons in the striatum, cerebral cortex, and hippocampus portions as it significantly reduced the numbers of nuclear pyknosis caused by cyclophosphamide. Thus, natural lutein could be considered a prospective immunomodulator agent for treating different inflammation-related immune conditions.

## Data Availability

All data generated or analyzed during this study are included in the manuscript.
